# Leveraging textual information for social media news categorization and sentiment analysis

**DOI:** 10.1371/journal.pone.0307027

**Published:** 2024-07-15

**Authors:** Mahmudul Hasan, Tanver Ahmed, Md. Rashedul Islam, Md. Palash Uddin

**Affiliations:** 1 Department of Computer Science and Engineering, Hajee Mohammad Danesh Science and Technology University, Dinajpur, Bangladesh; 2 Department of Computer Science and Engineering, Varendra University, Rajshahi, Bangladesh; Sejong University, KOREA, REPUBLIC OF

## Abstract

The rise of social media has changed how people view connections. Machine Learning (ML)-based sentiment analysis and news categorization help understand emotions and access news. However, most studies focus on complex models requiring heavy resources and slowing inference times, making deployment difficult in resource-limited environments. In this paper, we process both structured and unstructured data, determining the polarity of text using the TextBlob scheme to determine the sentiment of news headlines. We propose a Stochastic Gradient Descent (SGD)-based Ridge classifier (RC) for blending SGDR with an advanced string processing technique to effectively classify news articles. Additionally, we explore existing supervised and unsupervised ML algorithms to gauge the effectiveness of our SGDR classifier. The scalability and generalization capability of SGD and L2 regularization techniques in RCs to handle overfitting and balance bias and variance provide the proposed SGDR with better classification capability. Experimental results highlight that our string processing pipeline significantly boosts the performance of all ML models. Notably, our ensemble SGDR classifier surpasses all state-of-the-art ML algorithms, achieving an impressive 98.12% accuracy. McNemar’s significance tests reveal that our SGDR classifier achieves a 1% significance level improvement over K-Nearest Neighbor, Decision Tree, and AdaBoost and a 5% significance level improvement over other algorithms. These findings underscore the superior proficiency of linear models in news categorization compared to tree-based and nonlinear counterparts. This study contributes valuable insights into the efficacy of the proposed methodology, elucidating its potential for news categorization and sentiment analysis.

## Introduction

There has been significant and rapid advancement in information technology, leading to the emergence of social media as a dominant phenomenon. Social media provides an online platform for user-to-user interactions, including messaging, photo sharing, blog commenting, and status updates. Individuals extensively use prominent social networking sites such as Facebook, Instagram, and Twitter to express their opinions, making social media a valuable data source for sentiment analysis and text mining [1]. News dissemination through various social media platforms profoundly impacts our news consumption habits [2]. News organizations facilitate audience engagement by incorporating Facebook “share” and Twitter “retweet” buttons on their web pages, encouraging the natural human behavior of news sharing [3]. As digital technology has expedited sharing processes, social media’s widespread usage underscores the need to understand social media activities in society and the nation. The use of social media has surged in recent years, with Facebook having a global user base of 2.79 billion [4]. The platform’s widespread adoption allows users to freely express their viewpoints on diverse topics, generating vast amounts of valuable data [5].

News categorization and sentiment analysis from social media are motivated by several factors. Firstly, the vast amount of data generated on social media platforms necessitates efficient methods to sift through and categorize news articles and user-generated content. This is crucial for identifying relevant information amidst the noise, facilitating timely access to news updates, and enhancing information dissemination. Secondly, understanding public sentiment toward news events, products, or brands provides valuable insights for businesses, policymakers, and researchers. Sentiment analysis aids in gauging public opinion, detecting emerging trends, and guiding decision-making processes. Moreover, social media news categorization and sentiment analysis enhance the user experience by personalizing content delivery and recommending relevant news articles or products based on user preferences and sentiments. These tasks play a vital role in information management, communication, and decision-making in the digital age.

In recent years, there has been a notable trend in news categorization and sentiment analysis towards utilizing Machine Learning (ML) and Deep Learning (DL) techniques. ML algorithms have traditionally been employed for these tasks due to their effective handling of structured data. However, DL models have become powerful tools for extracting intricate patterns and representations from unstructured textual data. The utilization of both ML and DL in news categorization and sentiment analysis has shown good results, showcasing enhanced accuracy and scalability, thus driving further research and application in this domain. Due to the simplicity, interpretability, and efficiency in handling smaller datasets, ML is often preferred over DL in such real applications. ML models require less computational resources, making them more practical for real-world applications. Additionally, ML algorithms can offer comparable performance to DL models in certain scenarios with less complexity.

In this study, we focus on sentiment analysis of news texts shared on social media, assuming that individuals primarily share news related to their beliefs and interests. The proposed system aims to classify the news shared on social media to perform sentiment analysis. By categorizing news, we gain insights into the emotions of those who share it. Categorizing news into different topics, such as educational, political, entertainment, and technological news, has piqued users’ interest. However, the process of news classification can be labor-intensive. It involves extracting key features from news titles and using them to automatically classify news articles into predefined categories based on training data [6]. Sentiment analysis also has emerged as a popular field with numerous applications. Given these factors, there is a growing need to categorize news stories based on their tone and conduct mood analysis. For news categorization and sentiment analysis, in this paper we formulate some research questions as (*i*) Do sentiment analysis models identify the positive, negative, and neutral sentiments often seen on social media?, (*ii*) How can news articles and social media data be effectively categorized using ML algorithms?, and (*iii*) How do various preprocessing techniques, such as tokenization, stemming, and stop-word removal, affect the performance of ensemble models in news categorization tasks?

To get the answers to the questions, we propose an ML-based news categorization system employing ML algorithms, and we design a blending ensemble classifier and propose a string preprocessing pipeline to process the unstructured data. The proposed methodology can classify both labeled and unlabeled data that have valuable insights into the realm of text categorization. Employing K-fold cross-validation, the suggested approach enables businesses to collect user feedback, which, when combined with sentiment analysis, can provide valuable insights to understand customer interests and tailor offerings accordingly. Organizations can benefit from focusing on the most widely read news through effective news categorization. This study addresses the significant influence of social media in the context of sentiment analysis and news categorization. The key contributions of this study are summarized as follows.

- To perform sentiment analysis on social media text data, we process both structured and unstructured data to determine the polarity of individual sentences.
- We propose a string preprocessing technique that includes lowercase conversion, punctuation and stop-word removal, stemming, and lemmatization to make the dataset more ML-centric to get better classification performances.
- For classifying unlabelled news, we employ unsupervised ML algorithms capable of creating accurate labels for the unlabelled news, show a comparative analysis of supervised and unsupervised ML algorithms for news categorization, and find the best groups of algorithms that are more robust to news categorization.
- For enhanced news categorization, we develop an ensemble classifier blending Stochastic Gradient Descent (SGD) and Ridge classifier, termed SGDR, that utilizes proposed string processing techniques and outperforms benchmark ML classifiers.
- To verify the superiority of the proposed SGDR classifier, we employ the McNemar significant test to find the level of significance of the proposed method.

The remaining part of this paper is organized as follows: The materials and methods section provides a detailed explanation of the proposed system, working procedures, and its structural components. The result and analysis section discusses the proposed system analysis’s environmental configuration and experimental results. Finally, the conclusion section presents the concluding remarks and suggestions for future work to expand and further develop the proposed approach.

## Related works

Consequently, numerous ML-based models have been developed to recommend social media trends, often called sentiment analysis [7]. An insightful study of Neural Networks (NN), Support Vector Machine (SVM), Decision Tree (DT), Random Forest (RF), and Naive Bayes (NB) is done for the classification of news based on their accuracy. The experimental results have led to the conclusion that NB gives better results with an accuracy of 96.8% while the DT, NN, SVM, and RF provide accuracy of 83.2%,96.4%, 96.4%, and 94.1% respectively [6]. An effective sentiment analysis on 36,500 comments on Facebook, 36,500 tweets, and 36,500 online votes related to ‘iPhone’ shows accuracy waves from 79% to 87% for Artificial Neural Networks without using extra memory space for storing the intermediary data [8]. A hybrid technique combining RF and NB for three Twitter datasets (100, 250, 500 tweets) related to keywords- ‘Amazon,’ ‘Hachette’ along with six types of pre-processing techniques provides an accuracy of 95.62%, 92.39%, and 94.19% respectively [9]. The Bayesian version of the Multinomial NB classifier provides better and similar performance than the Multinomial NB classifier for text classification [10]. The SVM classifier provides good performance of 84.87% accuracy on mobile phone brands’ reviews on Amazon, categorizing the sentiments into joy, surprise, trust, disgust, sadness, fear, anger, and anticipation [11]. An empiric analysis of 200 research papers with an explanation of Deep Belief Networks, GRU, LSTM, RecNNs, RNNS, and CNNs, and their working mechanisms is presented in [12] concluding that LSTM performs better than the other DL-based techniques. An analysis of three ML-based algorithms, KNN, RF, and Logistic Regression (LR) for the BBC news text dataset proves that LR, along with the TF-IDF vector, is the best among the mentioned algorithms by obtaining 97% [13]. Time consumption has been reduced for big data analysis using canopy as a prepossessing technique for the k-means Clustering algorithm. Sabah et. al., [14] proposed and evaluated this technique on the Dental healthcare insurance news dataset based on the Hadoop Distributed File System (HDFS). The method can shrink the execution time from 68.657 to 60.75 seconds. Based on TextRank, a semantic clustering news keyword extraction technique named Semantic Clustering TextRank (SCTR) is evaluated in a Chinese news library. The experimental results give a maximum precision gain of 71%, FM 75%, and recall of 92%. A clustering method for categorizing news that combines Grid search based on Canopy with K-means clustering (KMGC-Search) provides scores 96%, 92%, 94%, 94%, and 95% for ARI, HS, CS, VM, and FM scores, respectively [15]. Sentiment analysis involves determining emotions and feelings through the analysis of text-based information. By scrutinizing people’s comments, likes, and shares on social media, sentiment analysis helps infer their overall sentiment towards a particular event or topic. It provides valuable insights into people’s opinions across various domains, including medical, social, and political realms [2, 16].

An insightful investigation has been done in using nine different classifiers BART-large, ELectra-small, XLM-RoB-ERTa-base, ALBERT-base-v2, DistilBERT, RoBERTa-large, RoBERTa-base, BERT-large, and BERT-base for three different datasets- COVID-19 Fake News Data, extremist-non-extremist dataset. For each of the three datasets, Qasim et al. [17] individually displayed and compared the classification performance of the nine methods. The investigation concludes best-performed models- RoBERTa-base for the COVID-19 Fake News Dataset, Bart-large for the COVID-19 English Tweets Dataset, and both BERT-base and BERT-large for the Extremist-Non-Extremist Dataset. Khosa et al. [18] proposed a hybrid model consisting of RF and SoftMax regression for news categorization results in 98.1% of accuracy for the BBC news dataset and 100% for the “business” news category. To identify false reports on COVID-19, the research project by Wani et al. [19] explores 8 ML techniques SVM, DT, LR, RF, KNN, Adaboost, NB, Neural Networks, and 5 DL classifiers- GRU, CNN, LSTM, BiLSTM, and RNN together with data preprocessing techniques. Amongst the ML classifiers, jointly KNN, multilayer perception, RF, and DL techniques CNN and BiLSTM are the most efficient for detecting false news, attaining an accuracy of 97%. A thorough investigation of text classification using ML, with a focus on news article classification, was conducted by Daud et al. [20] on the Reuters news dataset. The hyperparameter-optimized SVM was suggested in this study to classify news items into the appropriate categories and improved 20.814% accuracy. The optimized hyperparameter was determined based on the outcomes of SVM for different combinations of the parameters. The classification results are compared with the optimization results of other ML techniques like NB, KNN, LR, RF, and SGD. Rigorous research was conducted to identify fake reviews in [21]. A dataset of 68,000 review comments from the Google Play store and 512 end-user responses from the University of Science and Technology Bannu, Pakistan, were considered for the investigation. The investigated ML and DL classifiers provide a promising performance of 96% average accuracy, whereas the end-users accuracy was 44%. Khan et al. [22] provide a unique method for using a news sequential evolution model (NSEM) based on distributed representations to identify and analyze hot topic trends in streaming text data. To identify and visualize patterns in text data streams more accurately than current approaches, that uses DL techniques like word2vec models and Long Short-Term Memory (LSTM). Shafqat et al. [23] explored different ML algorithms like RF, DT, Kstar, Bayes Net, and NB for opinion mining of Politics and Inflation using the Roman Urdu dataset from Kaggle. The observation concludes RT is the best classifier among the investigated ones and in terms of execution time, NB exhibits superior performance. A novel Generic Algorithm-based approach for detecting false news on social networks is introduced in [24] alongside investigating different ML algorithms- LR, RF, NB, and SVM. Experiments were conducted on three datasets- the Kaggle fake news dataset, the LIAR dataset, and the FJP dataset. The suggested GA-based method outperforms the ML techniques by a small margin. Marwat et al. [25] suggested a SentiDeceptive technique that automatically divides end-user reviews into negative, positive, and neutral feelings to retrieve,e deceptive end-user rating information. It also recognizes online product evaluations based on crowd-user comments on social media. Their exploration included the steps- preprocessing the dataset, resampling the dataset, extracting features using TF-IDF and BOW from textual data, and implementing KFold and Shuffle Split validation. The results indicate that the best classifier for the suggested method is the Linear SVC classifier using a mix of BOW and SMOTE.

In the realm of data classification, [26] proposed a novel variant of the Bat Algorithm (BA) called the Improved Bat Algorithm (IBA). This approach refines the standard BA by enhancing its exploitation capabilities and mitigating the risk of being trapped in local minima. Another study [27] employed quasi-random sequences for population initialization rather than a random distribution to improve convergence and population diversity further. This study reviews various initialization methods used in Particle Swarm Optimization (PSO) that are based on quasi-random sequences (e.g., Halton, Torus) and pseudo-random sequence strategies (e.g., LCG, MCG). Genetic algorithm-based techniques have recently been adopted in Femtocell communication systems due to their inherent advantages. In a research study, the authors [28] presented a genetic algorithm-based technique designed to optimize coverage, power, and bit error rate, thereby enhancing Femtocell performance. Simulation results indicate that the genetic algorithm-based optimization technique effectively achieves superior performance in a Femtocell environment.

Existing methods have shortcomings compared to traditional ML models for news classification. DL requires vast amounts of labeled data and computational resources, making it resource-intensive and often impractical for small datasets. Additionally, those models lack interpretability, making it difficult to understand their decision-making process. Furthermore, existing methods are susceptible to overfitting, especially in scenarios with limited data or noisy datasets. Therefore, traditional ML models may offer more practicality, transparency, and generalizability for news classification tasks in certain contexts.

To show the performance of different research and compare it with our proposed method, we create Table 1 that shows the related dataset, methods and obtained individual research results. Our result is also included here to show the superiority of our proposed methodology.

**Table 1:** Details comparison of the related literature of news categorization.

Ref	Dataset	Technique	Score
[11]	Mobile Phone brands review on Amazon	SVM	SVM = 84.84%
[8]	36,500 Facebook Comments, 36,500 tweets, and 36,500 votes related to ‘iPhone’	ANN	ANN = 87%
[9]	Three Twitter datasets	Combined approach of RF and NB	Combined RF and NB = 95.62%
[13]	BBC news text dataset	LR with TF-IDF, KNN, RF	LR with TF-IDF = 97%
[6]	BBC news text dataset	NB, DT, NN, SVM, RF	NB = 96.8%
[14]	HDFS	Canopy with k-means Clustering	Reduction in execution time from 68.567 to 60.75
[17]	BBC news text dataset	Hybridization of RF and softmax function	Proposed model = 98.1%
[18]	Reuters news dataset	Hyperparameter optimized SVM, NB, KNN, LR, RF and SGD	Optimized SVM = 91.30%
[16]	A Chinese news dataset	SCTR and BERT	Precision 71%, FM %75 and Recall 92%
[15]	BBC news text dataset	K-means clustering, Spectral clustering, KMGC-Search	KMGC-Search = 96%, 92%, 94%, 94% and 95% for ARI, HS, CS, VM and FM score respectively.
SGDR (Proposed)	BBC news text dataset	Blending SGDR Classifier	Blending SGDR = 98.12%

## Materials and methods

**Fig 1:**
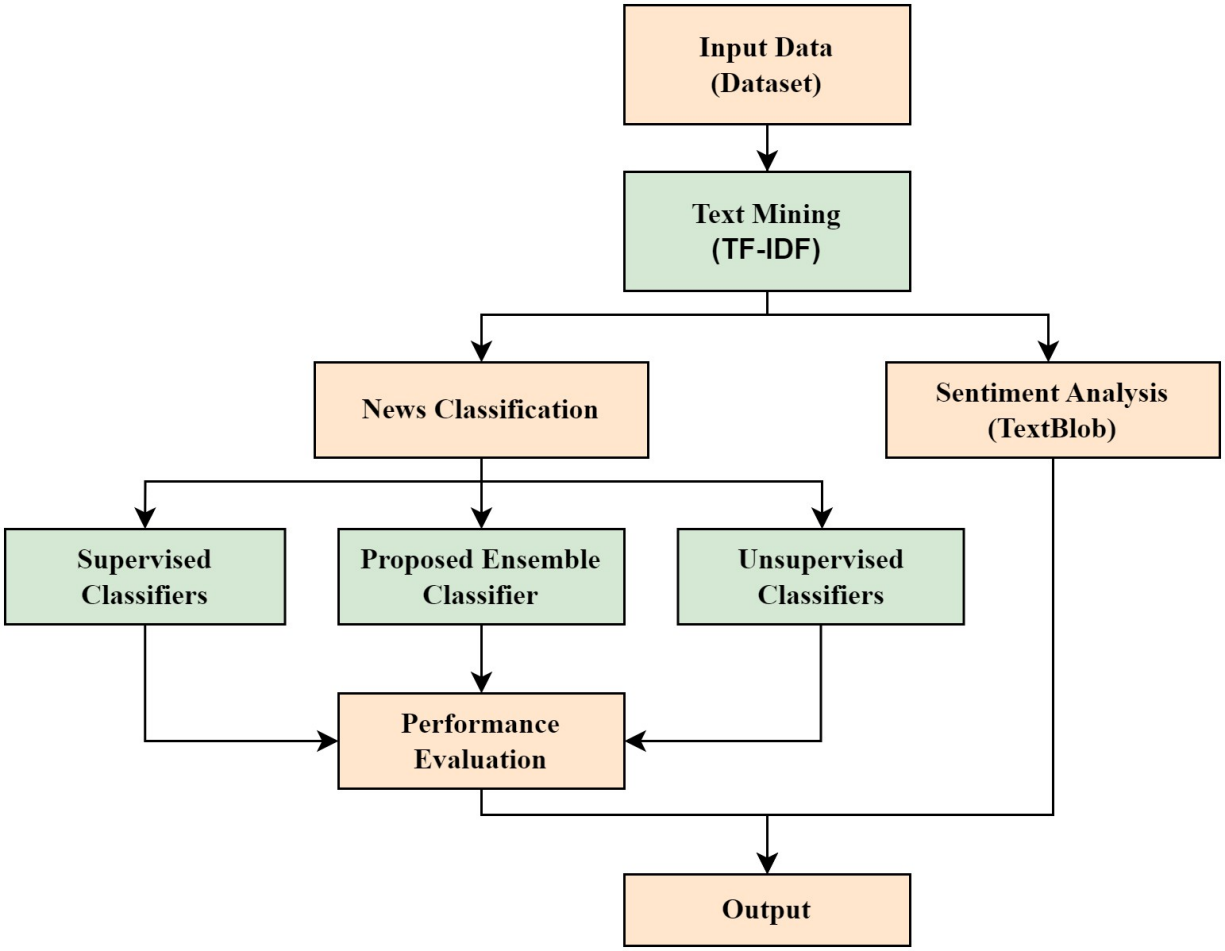
Methodology of news classification and sentiment analysis.

Sentiment analysis and news classification represent two prominent domains where ML algorithms are frequently employed. However, the classification accuracy achieved by ML-based systems exhibits substantial variability. While one system might yield superior accuracy, another could demonstrate inferior results. To address this issue, an approach has been introduced to streamline and simplify the processes of sentiment analysis and news classification. A succinct overview of this proposed approach is presented in Fig 1. The system has been fueled by data derived from the BBC-text dataset [29], encompassing 2225 news articles spanning five categories: “sport,” “business,” “politics,” “entertainment,” and “tech.” The Facebook API was leveraged to amass URLs for newspapers and corresponding news titles. This method, grounded in the BBC-text dataset, was subsequently applied to bdnews24 for sentiment analysis. The ensuing step involved text mining, where TF-IDF was employed to filter out extraneous words and demarcate semistructured from unstructured text. The text mining process facilitated news components’ classification and comprehensive mood analysis. For news classification, a suite of ML algorithms including SVM, K-Nearest Neighbours (KNN), DT, Adaptive Boosting (AB), Multinomial Naïve Bayes (MNB), LR, SGD, Ridge Classifier (RC), Non-Negative Matrix Factorization (NMF), and K Means Clustering (KMC) was harnessed. The performance of our proposed ensemble ML model exceeded that of prevailing ML models. In essence, this proposed methodology has ushered in a novel news article category and emerged as a source of news sentiment analysis.

### Dataset description

**Fig 2:**
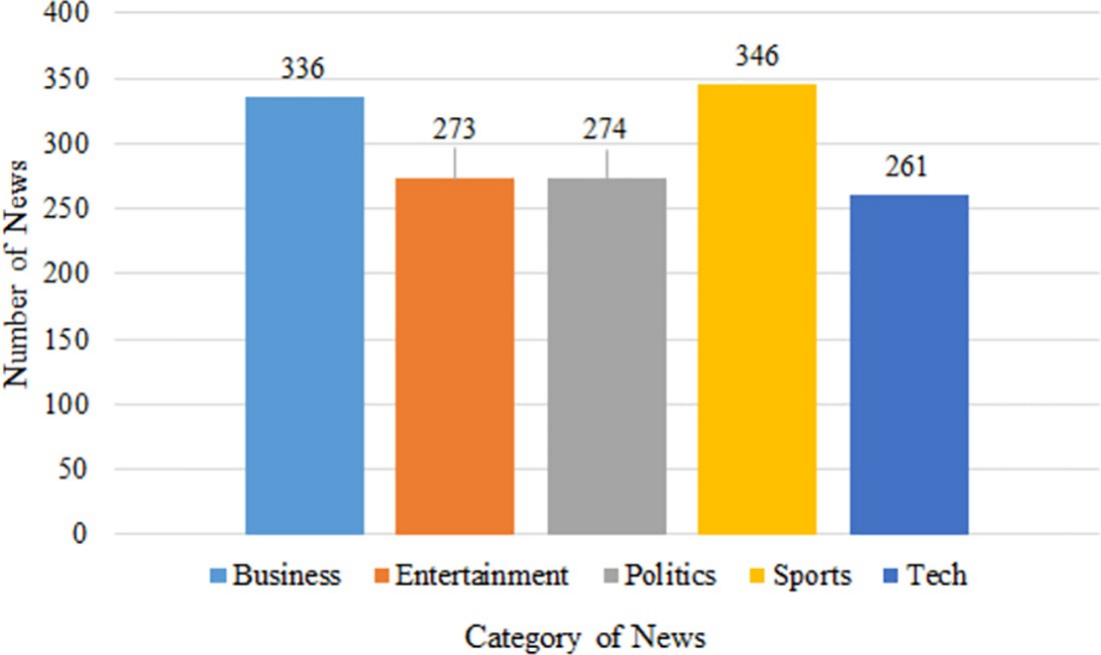
Category of news with news frequency.

In this study, the BBC-text dataset [29] served as the foundation for news categorization. Comprising a collection of 2225 news articles, the dataset encompasses five distinct news categories: ‘sport’, ‘business’, ‘politics’, ‘tech’, and ‘entertainment’, as depicted in Fig 2. Within this framework, sports-related news articles have been allocated to the ‘sport’ category, while news articles focusing on various organizations or services have been designated as belonging to the ‘business’ category. Similarly, news articles centered around political matters have been assigned to the ‘politics’ category, whereas those featuring themes of joy, cheer, gladness, pleasure, and entertainment find their place within the ‘entertainment’ category. News about science and technology have been fittingly grouped under the ‘tech’ category. Notably, an examination of the article distribution reveals a balanced dataset, indicating a lack of pronounced imbalances.

### Technical descriptions

#### Text mining

Text analytics and text mining are closely related. Text mining is the practice of extracting valuable information from text [30]. Text mining automatically extracts information from different documents that enclose structured textual content facts from the unstructured textual content. Term Frequency-Inverse Document Frequency (TF-IDF) has been used for text mining.

An information retrieval statistical measure method called TF-IDF assesses how relevant a term is to a document within a group of documents [31]. It is widely used in automated natural language processing and automated text analysis. To calculate the TF-IDF of any document, the first step is to tokenize the sentence to get the frequency of each word and the total quantity of words in the sentence as a whole [32]. The Term Frequency (TF) of a word is the ratio of the frequency of the word and the total quantity of words in the sentence.

$$\begin{array}{c} TF=\frac{Frequency\;of\;the\;word\;in\;the\;sentence}{Total\;number\;of\;words\;in\;the\;sentence} \end{array}$$ (1)

On the other hand, Inverse Document Frequency (IDF) is the ratio of the total number of sentences in a document and the number of sentences containing a particular word. So, IDF can be defined as,

$$\begin{array}{c} IDF=log\;\frac{Total\;number\;of\;sentences\;(documents)}{Number\;of\;sentences\;containing\;the\;word} \end{array}$$ (2)

#### Sentiment analysis

Sentiment analysis involves detecting and analyzing user opinions, attitudes, and emotions present in the text, categorizing them as positive, negative, or neutral [33]. Within Natural Language Processing, this task holds significant prominence [34]. Sentiment analysis is frequently applied to textual data or records to aid organizations, companies, and businesses in gauging the impact of their brand and products on consumers, ultimately fostering a deeper comprehension of customer preferences [35]. Numerous methods exist for assessing text sentiment [36]. The proposed system employs the TextBlob Python library to conduct sentiment analysis on news articles.

#### TextBlob

**Fig 3:**
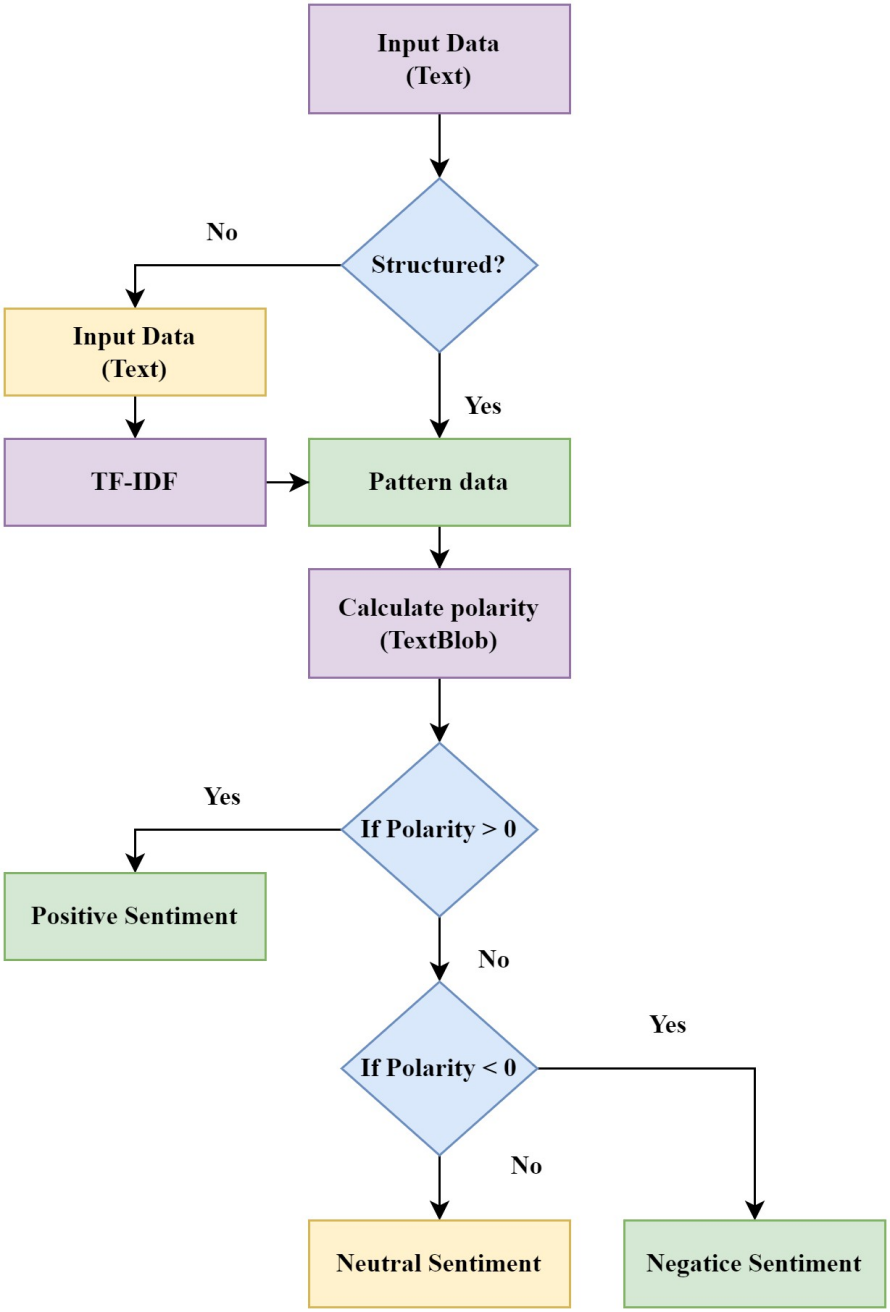
Flowchart of sentiment analysis.

TextBlob is one of the most robust Python libraries for processing textual data. It offers a user-friendly API that facilitates engagement with various common Natural Language Processing (NLP) tasks, including text classification, language translation, sentiment analysis, noun phrase extraction, part-of-speech tagging, and more [37]. Built on the foundation of the Natural Language Toolkit (NLTK), TextBlob provides an accessible interface to the capabilities of the NLTK library. Notably, TextBlob has been harnessed for sentiment analysis, enabling the computation of sentiment based on numeric values representing polarity and subjectivity. These numeric values indicate the extent to which a text is positive, negative, or neutral in sentiment. A polarity value of zero signifies a neutral sentiment, while values greater than zero denote positivity, and values less than zero indicate negativity [38]. The distinction between objectivity and subjectivity in a sentence is consequential. Objectivity refers to factual information, while subjectivity encompasses opinions, feelings, or judgments. Subjectivity values range from 0 to 1, gauging the mix of factual content and personal opinion in textual data. Higher subjectivity values signify a greater presence of personal opinion. In sentiment analysis, the operational procedure of the proposed system is outlined in Fig 3. Initially, the model is trained using predefined data. Incoming input is then assessed to ascertain whether it conforms to a structured format. If unstructured, TF-IDF eliminates stop words and generates patterned data. Subsequently, polarity and subjectivity are computed using TextBlob. The polarity is then examined, determining whether it is greater than zero, less than zero, or equal to zero. If polarity surpasses zero, the output reflects a positive sentiment; if it falls below zero, the output signifies negativity. Otherwise, a neutral sentiment is conveyed.

#### News classification

Text categorization, also known as text tagging or labeling, entails organizing sequential text into distinct categories. Employing NLP, a text classifier can automatically assess text, assign predefined tags, or categorize it based on its content [39, 40]. Text classification serves various purposes, including sentiment analysis, language identification, product or content tagging, spam detection, and more [41].

**Fig 4:**
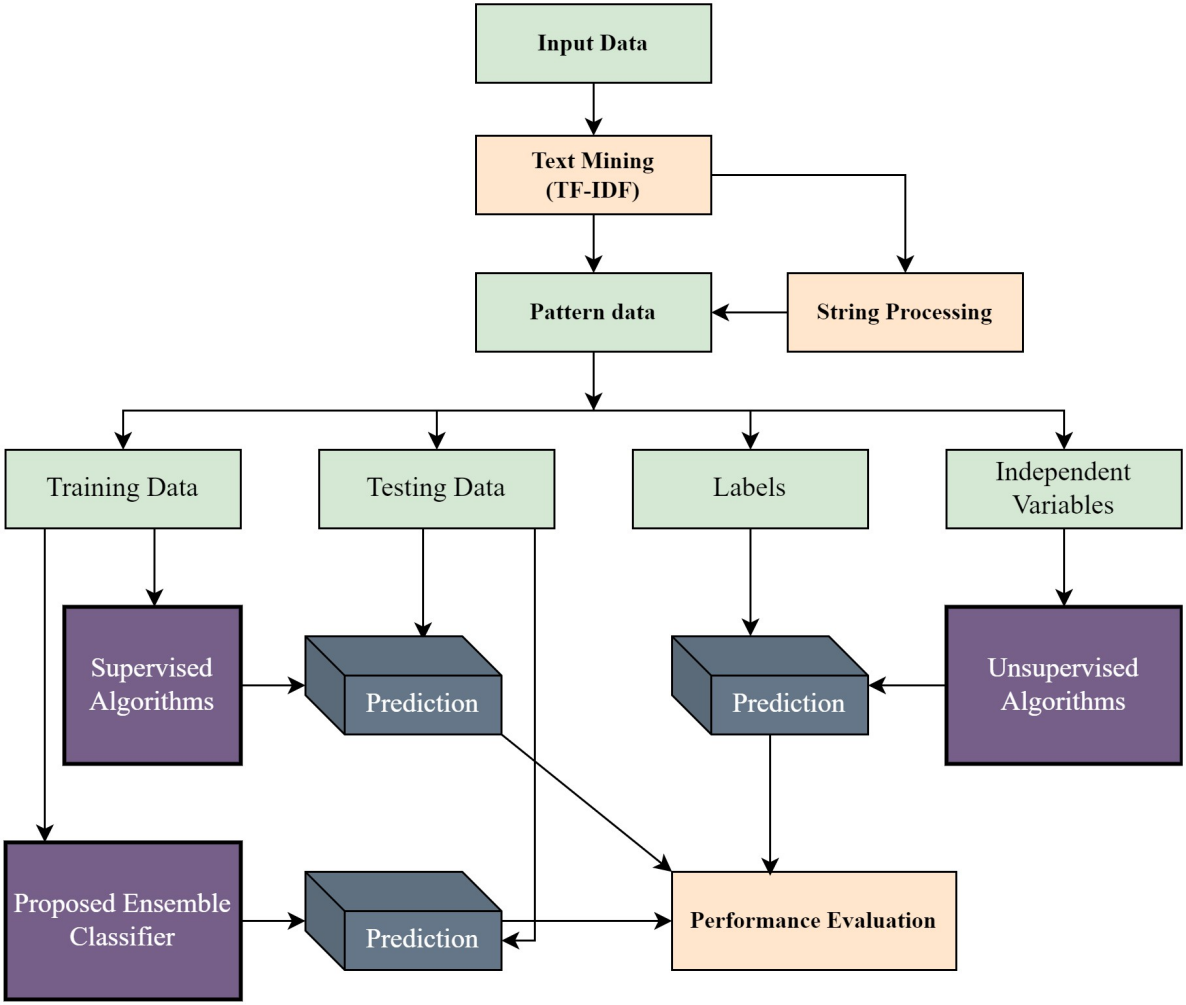
Top-down approach of news categorization using ML. Both supervised and unsupervised algorithms are used, and individual performance is analyzed.

ML and Ensemble ML algorithms have been employed in the proposed system for news classification. The procedure for news classification is illustrated in Fig 4. The model is initially trained using pre-existing data. The input dataset is assessed to determine whether the text is structured. TF-IDF transforms the data into a structured pattern if the text lacks structure. Following the analysis of patterned data, the proposed system individually employs algorithms to classify news articles. The classifier’s performance is evaluated through a classification report encompassing accuracy, precision, recall, and F1 score, providing a comprehensive assessment of the classifier’s effectiveness.

**Algorithm 1** String Processing

 **Input**: String (***S***)

 **Output**: Process String

1: **procedure** *String Processing*(***S***)

2:  Convert *s* ← to lowercase(***S***)

3:  Remove punctuation’s (***s***)

4:  Remove stop-words (***s***)

5:  Stemming (***s***)

6:  Lemmatisation (***s***)

7:  Process String ***s***

8: **end procedure**

#### String processing

We preprocess the string to enhance its suitability for training ML models. Following the classifier’s outcome, we subject the dataset to the string processing technique again. Subsequently, we reapply the classifier to the processed dataset, yielding heightened accuracy across all classifiers. The string processing procedure is outlined in Algorithm 1.

### Description of the ML classifiers

In the current research, we employ supervised and unsupervised ML algorithms over DL and Transfer Learning (TL) for several reasons. DL is also getting popular in different domains [42]. ML is chosen due to its adeptness at generalizing with limited labeled data, rendering it ideal for tasks with smaller datasets like BBC news categorization that we use in this research. Moreover, ML proves more pragmatic in resource-constrained environments than DL, which demands substantial computational resources. ML is sufficient for straightforward classification tasks characterized by well-defined features, whereas DL excels in discerning complex hierarchical patterns or processing unstructured data like images, audio, or text. Given the absence of pre-trained models and the significant computational resources and expertise required for TL fine-tuning, ML emerges as the preferred choice. The study’s focus on tabular data classification benefits from ML’s efficacy in achieving satisfactory performance with simpler models, making it the pragmatic choice for the research endeavor. Details of the ML algorithms are discussed below.

This research uses SVC, KNN, DT, AB, MNB, SGD, RC, LR-supervised ML algorithms and NMF and KMC unsupervised ML algorithms for news categorization. During classification, SVC creates a hyperplane to separate the classes [43, 44]. Previously, it converted each training sample into a feature vector, representing different classes and minimizing an optimization function associated with the hyperplane’s parameters while maximizing the separation between them. It reduces the errors by penalizing misclassified features without overly compromising classification accuracy. However, the effectiveness of SVC may decrease in the presence of highly noisy data [45]. As with SVC, the KNN algorithm works based on the distance of the data points, especially in clustering and classifying data according to similarity [46]. KNN groups similar data points by comparing their characteristics using class labels and feature vectors [13, 47]. In text categorization, texts are represented as spatial vectors, and the algorithm calculates similarity scores between the training texts and a new text sample. Based on these similarities, the K most similar neighbors are selected to determine the class of the new sample. The process involves transforming incoming and training texts into feature vectors, comparing these vectors to assess similarity, and then choosing the KNN to classify the incoming text.

As with the distance-based SVC and KNN, the tree-based DT relies on a set of if-then-else decision rules to learn patterns from data and approximate even complex functions such as sine curves [48]. DT constructs a model represented as a tree structure, using decision nodes and leaf nodes [49]. Decision Nodes are employed to make pivotal decisions, which are then branched into various routes. Conversely, Leaf Nodes correspond to the final decision outputs [50]. As a bosting architecture, the AB classifier uses multiple classifiers on the same training dataset, improving its performance iteratively [51]. This approach combines these classifiers into a powerful final model, assigning weights to each.

From the perspective of probability-based classification, the MNB classifier determines document labels by first calculating each class’s prior probability, influenced by class distribution in the training set. It then refines these probabilities based on the document’s words [52, 53]. Classification is achieved using the Maximum a posteriori (MAP) rule, choosing the class with the highest combined probability. Parameter estimation during training uses a smoothing technique to counteract zero probabilities [10, 54], adjusting word and class occurrence counts to ensure reliable estimation under sparse data conditions. Similar to the probability-based MNB algorithm, LR computes the probability of an outcome variable to establish a correlation between a dependent (target) variable and one or more independent variables [55, 56]. The output of the target variable is represented through binary values, specifically 0 and 1. LR employs a sigmoid function for outcome prediction, yielding a value from 0 to 1. When the sigmoid function’s output is 0.5 or greater, it’s interpreted as 1; conversely, an output value less than 0.5 is interpreted as 0.

In the optimization and regularization perspective, SGD minimizes the objective function, such as a loss function that calculates the discrepancy between the expected and actual values [57]. It is a variation of the gradient descent algorithm. The main characteristic of SGD is that, rather than utilizing the complete dataset, it changes the model’s parameters at each iteration by calculating the gradient of the loss function from either a single sample or a small batch of samples. On the other hand, the Ridge Classifier includes a penalty term with the cost function to prevent overfitting [58]. It is an extension of the linear SVM classifier that introduces a regularization component called Ridge Regression or L2 regularization. In this study, we formulate it to tackle multiclass classification tasks using techniques like one-vs-one and one-vs-rest strategies.

**Fig 5:**
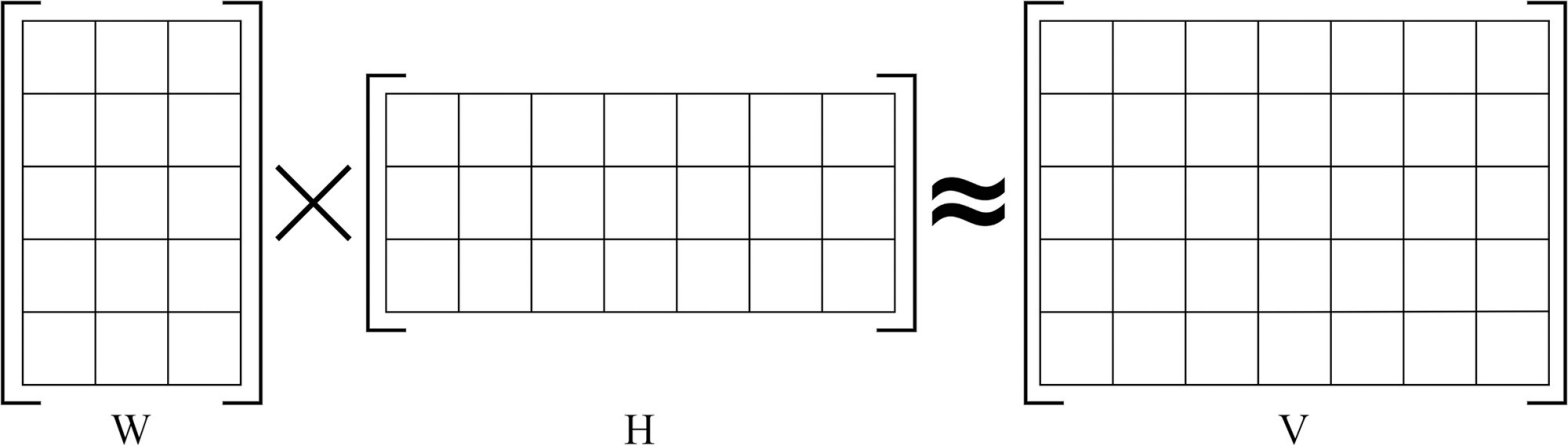
An illustration of NMF.

Additionally, we use two unsupervised techniques, namely NMF and KMC. NMF is designed to decompose high-dimensional vectors into lower dimensions while retaining non-negativity in both the lower-dimensional vectors and their coefficients. Unlike traditional matrix factorization methods, NMF factors non-negative matrices into two non-negative matrices, which approximate the original matrix. This technique finds utility in topic modeling and dimensionality reduction. Specifically, in topic modeling, the input consists of normalized TF-IDF data. We denote the input matrix as “A.” Following the factorization process, matrix “V” splits into two constituent matrices: “W” and “H.” in Fig 5.

Like NMF, KMC is also a clustering algorithm; it partitions an entire dataset into *K* clusters, each characterized by a distinct centroid [59]. These centroids function as representatives of their respective clusters. The primary objective of this algorithm revolves around minimizing the distances between data points and their associated clusters [60]. The KMC algorithm unfolds in two primary phases. An iterative technique determines the optimal number of K center points or centroids. Subsequently, the algorithm identifies the closest k-center for each data point and assigns the data point to the corresponding cluster. The efficacy of the KMC algorithm rests on how efficiently the clusters are formed. Usually, the Elbow method is used to determine the optimal number of clusters K.

### Proposed blending SGDR

Blending is a method of ensemble ML in which a model is trained to optimize the predictions of various models that make up the ensemble [61]. Blending, in this sense, is synonymous with stacking or stacked generalization. The main target of the ensemble is to reduce the variance and prevent over-fitting [62]. In the proposed blending SGDR, we use one optimization method SGD and one regularization ML model RC. The proposed blending SGDR can classify more accurately than the benchmark ML models and has the capability of handling overfitting.

SGD is an iterative optimization algorithm commonly used to train ML models. It aims to minimize the loss function by adjusting the model parameters in the direction that reduces the loss. Unlike traditional gradient descent, which computes the gradient using the entire dataset, SGD randomly selects a subset (mini-batch) of the data for each iteration. This introduces randomness and noise into the gradient estimation, which can help escape local minima and speed up convergence. The equation for updating the model parameters in each iteration of SGD is given in Eq (3).

$$\begin{array}{c} \theta_{t+1}=\theta_{t}-\eta .\nabla J\left(\theta_{t},x_{i},y_{i}\right) \end{array}$$ (3)

Where:

- *θ*_*t*_ represents the model parameters at iteration *t*.
- *η* is the learning rate, controlling the step size of the parameter updates.
- *J*(*θ*_*t*_, *x*_*i*_, *y*_*i*_) is the loss function for a single data point (*x*_*i*_, *y*_*i*_)*t*.
- ∇*J*(*θ*_*t*_, *x*_*i*_, *y*_*i*_) is the gradient of the loss function concerning the model parameters evaluated at *θ*_*t*_ and for the data point (*x*_*i*_, *y*_*i*_).

The key idea of SGD is to update the model parameters based on the gradient of the loss computed using only a small subset of the data, which makes it computationally efficient and able to handle large datasets.

The blending ensemble follows the below steps:

1. Sets for viz-training and sets for validation are segmented from the train set.
2. The training set of data is used to fit the model.
3. For the validation purpose of the model, predictions are made on the validation and test sets.
4. A new model is created using the validation set and its predictions as features.
5. The test and meta-features are subject to final predictions using this model.

**Fig 6:**
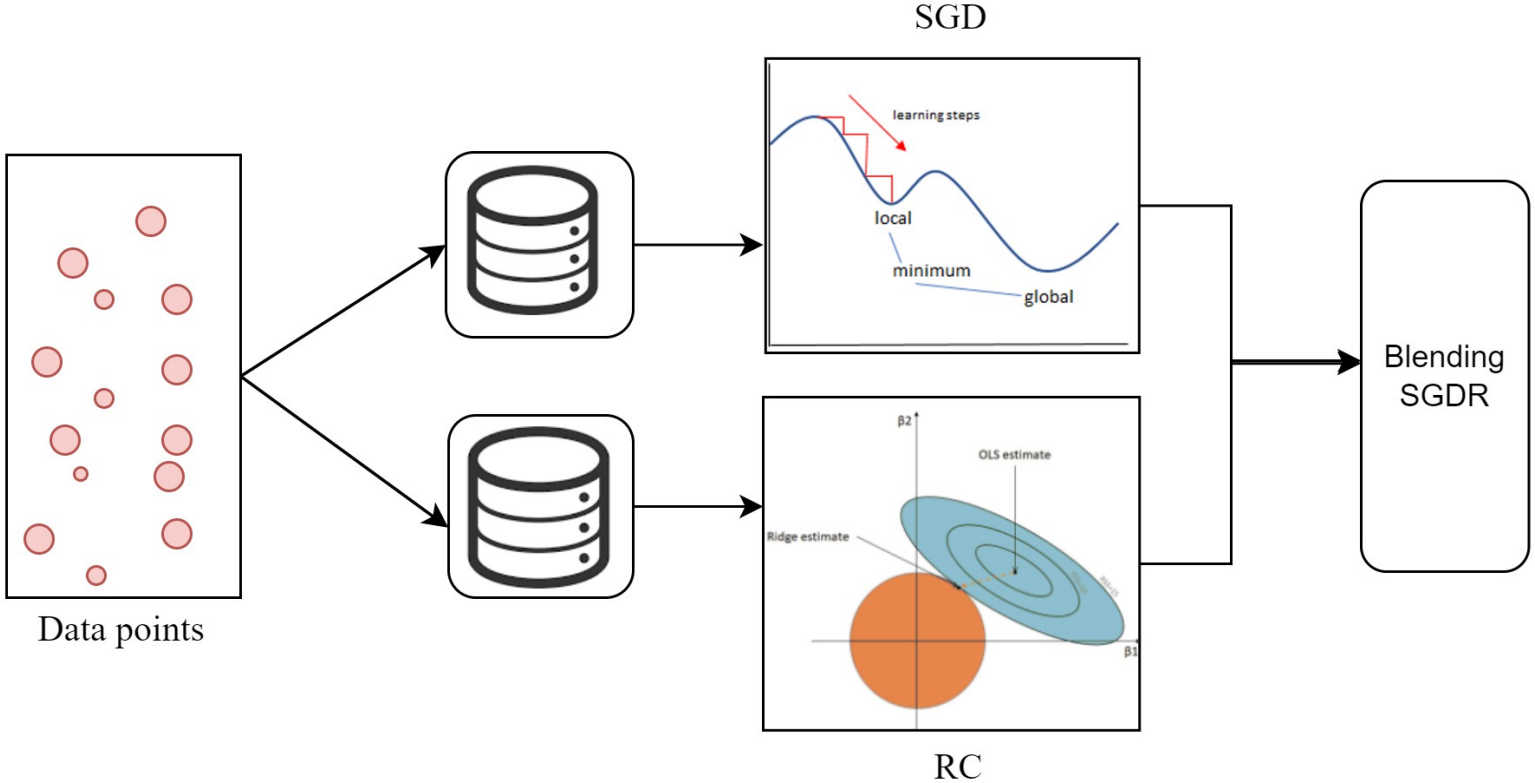
Block diagram of proposed blending SGDR algorithm.

Our proposed ensemble blending SGDR is represented in Fig 6 and Algorithm 2.

**Algorithm 2** Blending SGDR, which builds upon two algorithms including SGD, RC

 **S**: Dataset

 **M** : The number of classifiers (***M*** = *[****SGD***, ***RC****]*)

 **R** : The parameter determining the proportion of samples that need to be replaced

 **L** : The parameter used to control the distance from the synthetic samples to the training samples

 **Output**: Predicted output of the testing dataset

1: **procedure** *Ensemble_SGDR*(***S***)

2:  **for** i in *M* **do**

3:   Get a copy of the dataset *D*_*i*_ = *D*

4:   Get several training samples from the dataset and define it as P.

5:   **for** j in *P* **do**

6:    Train *M*_*i*_ by *P*_*i*_

7:   **end for**

8: Return new data from the output of *M*_*i*_

9:  **end for**

10: **end procedure**

### McNemar test

Statistical McNemar test is used in ML to evaluate the significance of performance differences between two classification models [63]. This is especially useful for cases where both models are employed for identical instances. The method is beneficial for processing paired or matched data because it makes comparing the classifications produced by two distinct models easier. The test involves constructing a 2 × 2 contingency table, where *b* is the count of instances misclassified by the first model but correctly by the second, and *c* is the count of instances misclassified by the second model but correctly by the first. The McNemar test statistic is calculated using the formula:

$$\chi^{2}=\frac{{\left(b-c\right)}^{2}}{b+c}$$

Then, the statistical technique is followed by a chi-squared distribution with 1 degree of freedom. The statistically significant classification model can be determined by exploiting the comparison between the computed *χ*^2^ value and the value derived from the chi-squared distribution. The McNemar test empowers decision-making regarding algorithm selection with the assistance of the models’ relative performance.

## Results

**Fig 7:**
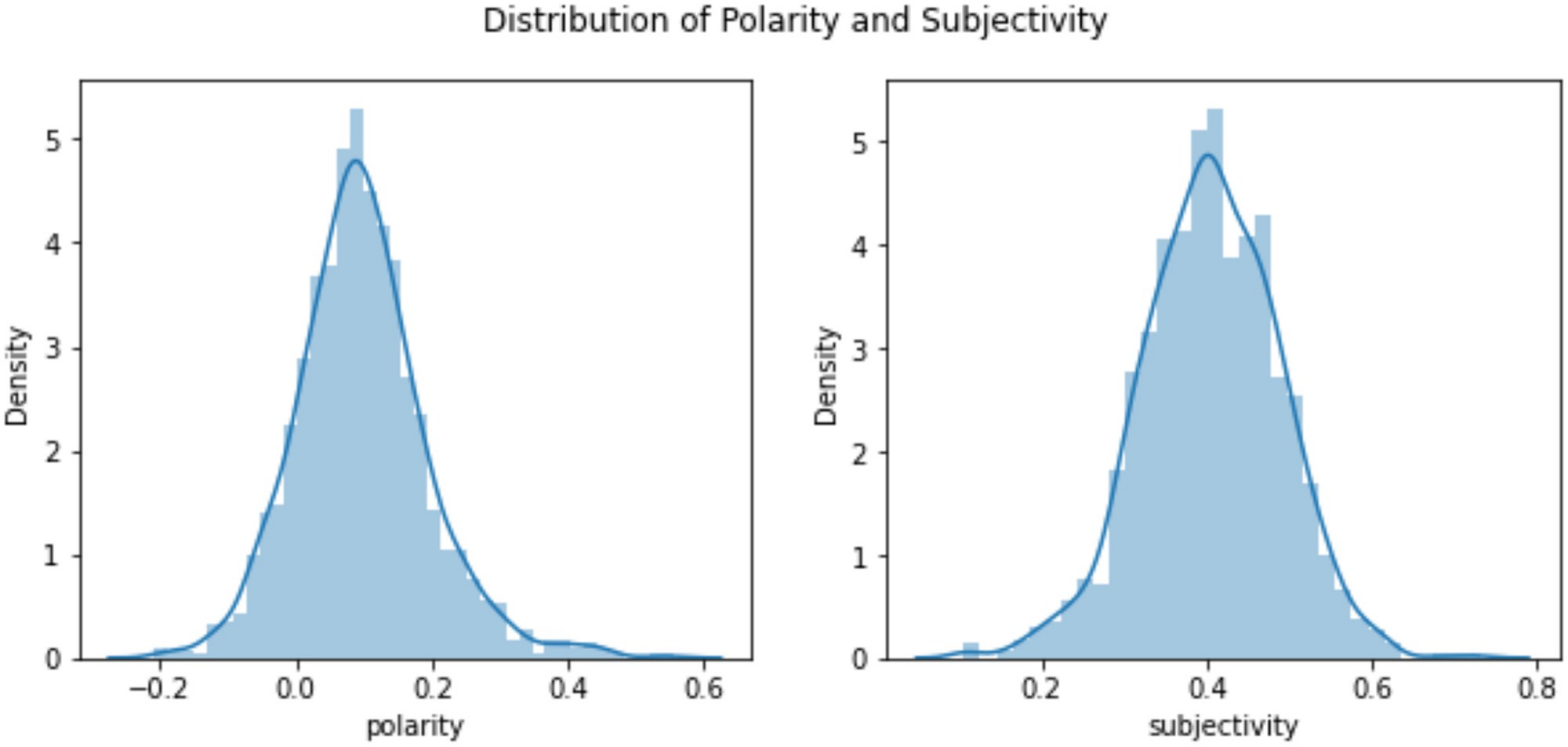
Polarity and subjectivity of BBC news dataset.

This research aims to find the sentiment of the text using TextBlob and categorize the news using existing ML and proposed ML models. Firstly, we explore the dataset to understand it clearly. We calculate the polarity and subjectivity of the dataset in Fig 7, which indicates that the dataset is stable in terms of its polarity and subjectivity, which helps the classifiers to make more accurate decisions. Polarity is biased to positive polarity, and most of the text’s subjectivity is 0.4.

### Sentiment analysis

We find the sentiment of the dataset using TextBlob. Table 2 shows the sentiment of three selected news headings with their polarity. The first heading, “The second trial is a visceral reckoning over Trump,” shows 0.0 polarity and is identified as a neutral sentiment. The second heading, “Biden bars Trump from receiving intelligence briefings,” shows -0.2 polarity, which shows negative sentiment. Similar to the first heading, the third sentence, “Watchdogs appointed by Trump pose a dilemma for Biden,” shows 0.0 polarity, which is also a neutral sentiment. Other news headings are also classified into different sentiment statuses using TextBlob. There may be many unnecessary words in the text for ML analysis. TF-IDF algorithm helps remove unnecessary words, sort the data, and extract the keywords from the text. TF-IDF can rank articles in order of relevance. The highest scoring words of a document are the most relevant words to that document; therefore, that word can be considered keywords for that document [64].

**Table 2:** Sentiment analysis.

News Title	Polarity	Sentiment Analysis
The second trial is a visceral reckoning over Trump	0.0	Neutral Sentiment
Biden bars Trump from receiving intelligence briefings	-0.2	Negative Sentiment
Watchdogs appointed by Trump pose a dilemma for Biden	0.0	Neutral Sentiment

### News categorization

Different ML algorithms are used to categorize the news article, and the performance of each algorithm is tabulated accordingly. We divide the algorithms according to their nature and show the results pairwise. The results of news categorization obtained by the different group classifiers are below.

### Results of hyperparameter tuning of the classifiers

In Table 3, we present the related hyperparameter and the values of the hyperparameter found by GridSearchCV search during model development for classification. These hyperparameter values help the classifiers to achieve maximum accuracy in the dataset.

**Table 3:** Hyperparameters of the classifiers and the suitable values obtain by GridSearchCV.

Method	Hyperparameter with values
SVM	C = 10, gamma = 0.001, kernel=‘rbf’, verbose = False
KNN	algorithm=‘auto’, metric=‘minkowski’, n_neighbors = 5, p = 2,weights=‘uniform’
LR	n_jobs = None, penalty=‘l2’,random_state = None, solver=‘newton-cg’, verbose = False
NB	var_smoothing = 0.045
DT	max_depth = 2, min_samples_leaf = 50, random_state = 42
AB	learning_rate = 0.01, n_estimators = 38
SGD	loss=“hinge”, penalty=“l2”
RC	alpha = 0.02, normalize = True
KMC	n_clusters = 5, init = ‘k-means++’, algorithm = ‘full’, random_state = 101
NMF	n_components = 5, init=‘nndsvda’, solver = ‘mu’, beta_loss = ‘kullback-leibler’, l1_ratio = 0.5, random_state = 101
Blending SGDR	loss=“hinge”, penalty=“l2”, alpha = 0.025, normalize = True

#### Performance of the distance-based ML classifiers

**Fig 8:**
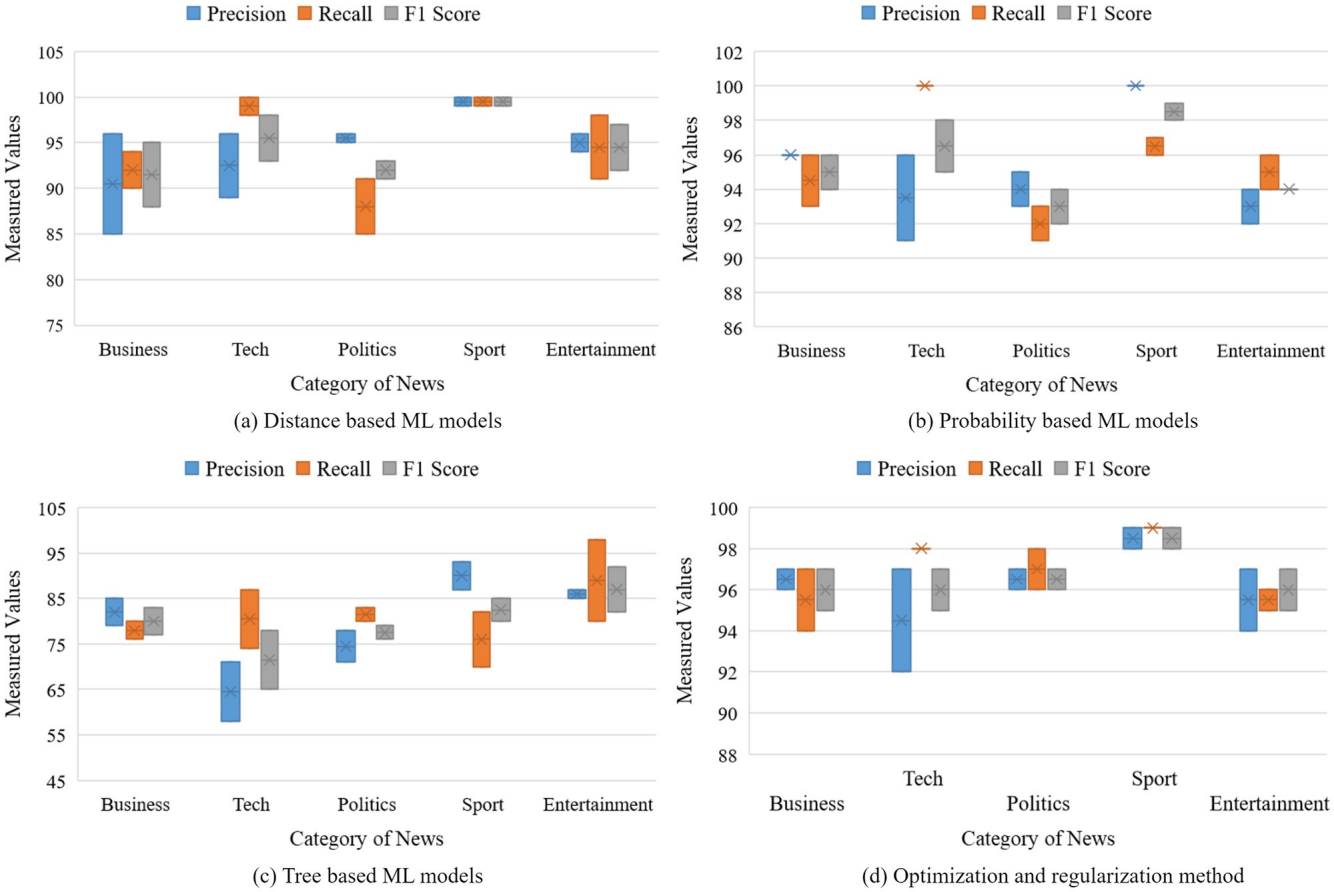
Range of the classification metrics for individual news category using ML classifiers.

Table 4 comprehensively evaluates the performance of distance-based algorithms applied to news categorization. The algorithms evaluated include SVM and KNN. For each algorithm, precision, recall, F1 score, Cohen’s Kappa coefficient, and accuracy (Acc) metrics are reported across various news categories, including Business, Tech, Politics, Sport, and Entertainment. Both SVM and KNN demonstrate high levels of accuracy, but SVM is somewhat more precise at 97% and KNN at 93%. When classifying sports news, the algorithm’s performance regarding precision, recall, and f1 score is unrivaled. Performance scores for sports news articles are almost 100% for SVM and 99% for KNN. For the classification of business news, both classifiers perform poorly. In this respect, SVM outperforms competing classifiers. Fig 8(a) displays various news classification methods’ precision, recall, and f1 score. The gap between the effectiveness of the two algorithms for various news categorizations is depicted by each bar. In terms of overall performance, the distance-based ML algorithm achieves 88% to 100% f1 score, 100% precision, and 85% to 100% recall.

**Table 4:** Performance of distance-based algorithms to news categorization.

Algo	News Category	Precision	Recall	F1 Score	Cohen Kappa	Acc
SVM	Business	96	94	95	96	97.08
	Tech	96	100	98		
	Politics	95	91	93		
	Sport	100	100	100		
	Entertainment	96	98	97		
KNN	Business	85	90	88	91	93.33
	Tech	89	98	93		
	Politics	96	85	91		
	Sport	99	99	99		
	Entertainment	94	91	92		

#### Performance of the probability-based ML classifiers

For a better analysis of news categorization, we have also investigated probability-based algorithms, LR and NB, on the BBC dataset. The classification report signifies that the accuracy rates of LR and NB are both very high. More precisely, LR generates 96% accuracy while NB produces 95%. Among all the news categories, both the LR and NB magnificently perform well. The NB provides scores of 100%, 96%, and 98% for precision, recall, and F1 scores, respectively. On the other hand, LR also performs very similarly to NB, providing scores of 100% for precision, 96% for recall, and 98% for F1 score. Both classifiers perform mediocrity when it comes to classifying other sorts of news. The comparison of the precision, recall, and f1 of these probability-based classifiers are demonstrated in Fig 8(b). Each bar represents the difference between the performance of two algorithms for different news categorizations. It can be observed from the performance Table 5 that LR performs better than rival classifiers, providing 96% overall accuracy in this regard.

**Table 5:** Performance of probability-based algorithms to news categorization.

Algo	News Category	Precision	Recall	F1 Score	Cohen Kappa	Accuracy
LR	Business	96	96	96	95	96.32
	Tech	96	100	98		
	Politics	95	93	94		
	Sport	100	96	98		
	Entertainment	92	96	94		
NB	Business	96	93	94	94	95.10
	Tech	91	100	95		
	Politics	93	91	92		
	Sport	100	97	99		
	Entertainment	94	94	94		

#### Performance of the Tree-based ML Classifiers

We have adopted the tree-based algorithms DT and AB for categorizing news. However, the performance of these methods is displeasing. The precision score of different sorts of news for DT ranges from 71% to 85%, where it is 71% to 93%. Though the recall score of entertainment news categorization for AB is quite high 98%, the other sort of news classification is extremely poor. The recall score of the news categorization for DT ranges from 80% to 87%. The F1 score varies between 79% to 85% for DT and 76% to 92% for AB. The bars illustrated in Fig 8(c) describe the precision, recall, and f1 scores of DT and AB algorithms. Though the investigated tree-based algorithms perform poorly, as described in Table 6, DT surpasses AB, providing 82% and 72% of overall accuracy.

**Table 6:** Performance of tree-based algorithms to news categorization.

Algo	News Category	Precision	Recall	F1 Score	Cohen Kappa	Acc
DT	Business	85	80	83	77	82.59
	Tech	71	87	78		
	Politics	78	80	79		
	Sport	87	82	85		
	Entertainment	85	80	82		
AB	Business	79	76	77	73	78.23
	Tech	58	74	65		
	Politics	71	83	76		
	Sport	93	70	80		
	Entertainment	87	98	92		

#### Performance of the optimization and regularization method

We have also adopted optimization and regularization ML techniques SGD and RC for news categorization. The performance of these methods is narrated in Table 7. Precision, recall, F1 score, Cohen’s Kappa coefficient, and Acc are meticulously documented for each algorithm and news category pairing pairing. These metrics are vital indicators of the algorithms’ ability to categorize news articles within distinct thematic domains accurately.

**Table 7:** Performance of optimization and regularization method to news categorization.

Algo	News Category	Precision	Recall	F1 Score	Cohen Kappa	Acc
SGD	Business	97	97	97	94	97.29
	Tech	97	98	97		
	Politics	96	96	96		
	Sport	99	99	99		
	Entertainment	94	95	95		
RC	Business	96	94	95	95	97.92
	Tech	92	98	95		
	Politics	97	98	97		
	Sport	98	99	98		
	Entertainment	97	96	97		

In the evaluation, SGD demonstrates notable prowess across all categories, boasting high precision (ranging from 92% to 99%), recall (ranging from 94% to 99%), F1 score (ranging from 95% to 99%), Cohen’s Kappa coefficient (94%), and accuracy (ranging from 97.29% to 97.92%). Particularly noteworthy is its exceptional performance in the Sports category, where it attains near-perfect scores across all metrics.

On the other hand, while showcasing commendable performance, RC exhibits slightly lower metrics than SGD in certain categories and measures. Nonetheless, RC maintains a robust performance profile across all categories, showcasing notable strengths in Politics and Sports classifications.

#### Performance of the unsupervised ML algorithms

**Fig 9:**
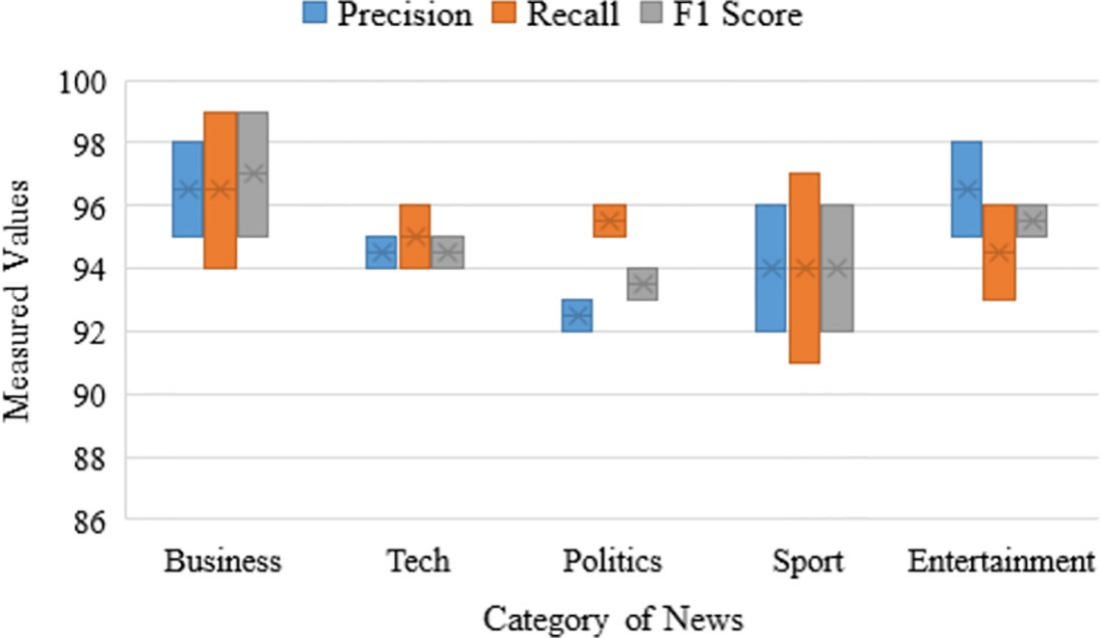
Range of the classification metrics for individual news category using unsupervised ML classifiers.

We have further explored unsupervised algorithms NMF and KMC for a more thorough analysis of news categorization. From Table 8 and Fig 9, the algorithms perform well in terms of precision, recall, and f1 score, but the NMF dominates the K-means Clustering in categorizing every sort of news.

**Table 8:** Performance of unsupervised algorithms to news categorization.

Algo	News Category	Precision	Recall	F1 Score	Cohen Kappa	Acc
NMF	Business	98	99	99	91	96
	Tech	95	96	95		
	Politics	93	96	94		
	Sport	96	97	96		
	Entertainment	98	93	95		
KMC	Business	95	94	95	90	94
	Tech	94	94	94		
	Politics	92	95	93		
	Sport	92	91	92		
	Entertainment	95	96	96		

For NMF, precision ranges from 93% to 98%, recall ranges from 93% to 99%, F1 Score ranges from 94% to 99%, Cohen’s Kappa coefficient remains at 91%, and accuracy ranges from 95% to 96%. Notably, NMF demonstrates particularly strong performance in the Business and Entertainment categories. On the other hand, KMC exhibits precision between 92% and 95%, recall between 91% and 96%, F1 Score between 92% and 96%, Cohen’s Kappa coefficient remains at 90%, and accuracy ranges from 94% to 94%. While KMC performs consistently across most categories, its performance is slightly lower than NMF, especially in the Politics and Sport categories. Both NMF and KMC showcase commendable performance in news categorization tasks, with NMF demonstrating a slightly stronger performance across most metrics and categories.

### Performance after applying string preprocessing algorithm

**Fig 10:**
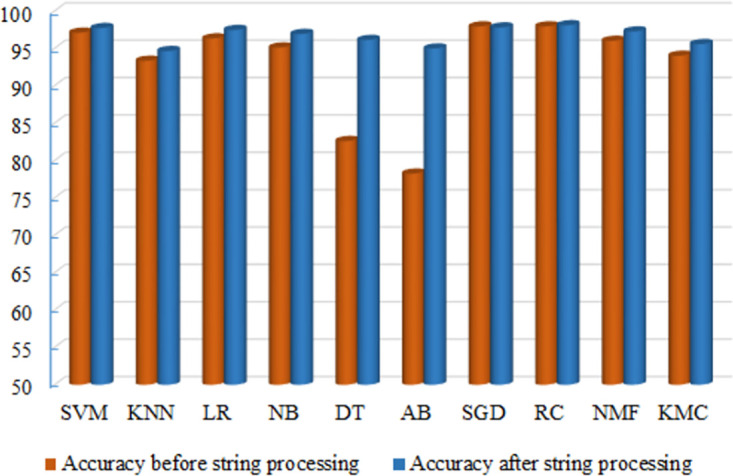
Comparison of the accuracy of the algorithms before and after string processing.

The algorithms’ performance increases after applying the string processing technique in this study, as evident from both the individual algorithm results and the collective outcomes listed in Table 9. Notably, every algorithm’s performance undergoes significant changes, underscoring the efficacy of the string processing algorithm. Furthermore, we have introduced a superior blending ensemble with an impressive accuracy of 98.12%. Detailed performance metrics are in Table 9. A comparison between the accuracy of algorithms before and after the implementation of the string preprocessing method is depicted in the bar chart illustrated in Fig 10. Across all scenarios, algorithm performance has a discernible enhancement, with the most substantial accuracy improvements observed in DT and AB. Specifically, DT’s accuracy climbs from 82.59% to 96.11%, while AB experiences an increase from 78.23% to 94.97%. Remarkable performance enhancements are also observed with other methods. These observations conclusively demonstrate the significant impact of the string processing algorithm on the classifiers’ performance.

**Table 9:** Performance of the classifiers after applying the string preprocessed techniques.

Algo	Accuracy	Algorithm	Accuracy
SVM	97.71	AB	94.97
KNN	94.63	SGD	97.79
LR	97.45	RC	98.08
NB	96.91	NMF	97.25
DT	96.11	KMC	95.56
Blending SGDR		98.12	

#### Top word calculation using unsupervised algorithm

**Fig 11:**
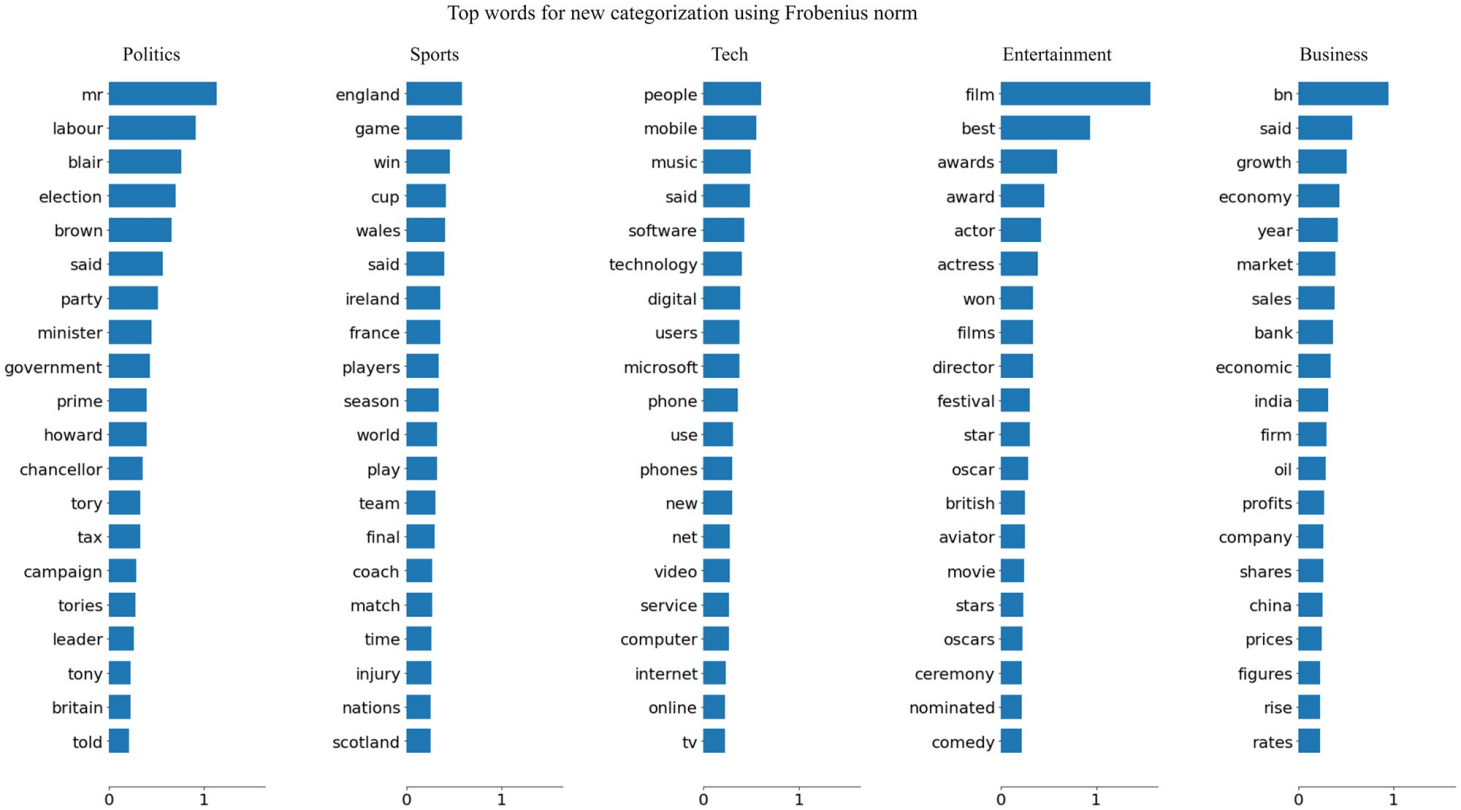
Tops words for each news category.

Using the best-unsupervised ML model NMF and TF-IDF, we calculate the top worlds responsible for each news categorization. The words’ impact is in Fig 11. The words indicate thahumans’he thinkinabouttcategorizinghe news is similar to the Mapproaches. The relevant words of the news category are listed in a decreasing other than their precedence in news categorization.

### Significant test

#### McNamr test

We also use McNemar’s Test to determine significance. McNemar’s Test generally compares two classifiers and determines how much one classifier is more significant than the other. As such, we compare Blending SGDR with SVM, KNN, NBC, DT, LR, AB, SGD, RC, NMF, and KMC ML algorithms. Table 10 shows the McNemar test result of the proposed SGDR compared to other algorithms. The hypothesis model in the McNemar test is 1% significant if the P-value is less than 0.01 and 5% significant compared to other models if the P-value is less than 0.05 and greater than 0.01. The result shows that the P-value for KNN, DT, and AB is less than 0.01 means SDGR is 1% significant, and for the other algorithms, the P-value is greater than 0.01 and less than 0.05. In this case, SGDR is 5% significance compared to the algorithms that show the statistical significance of the proposed method.

**Table 10:** McNemar test result of proposed SGDR compared to other algorithms.

ML Algorithm	P-value
SVM	0.02015
KNN	0.00475
LR	0.02941
NB	0.02516
DT	0.00015
AB	0.00012
SGD	0.01952
RC	0.01982
NMF	0.03301
KMC	0.03290

#### 10 Fold cross-validation result of the algorithms

We check 10-fold cross-validation results to assess model performance robustly. It divides the dataset into 10 subsets, iteratively training and testing the model on different folds. The result shows that the performance of the classifiers is unbiased, has less variance, and has no overfitting. The results are in Table 11.

**Table 11:** 10-Fold cross-validation accuracy of the algorithms.

Algo	F-1	F-2	F-3	F-4	F-5	F-6	F-7	F-8	F-9	F-10	Avg.
SVM	98	98	98	95	97	97	97	97	96	97	97
KNN	91	96	95	91	94	92	97	94	89	93	93.2
LR	98	98	98	96	97	97	98	97	97	98	97.4
NB	97	97	99	97	96	95	99	97	96	96	96.9
DT	83	85	76	85	85	82	82	80	86	81	82.5
AB	72	74	76	71	73	70	71	71	78	77	73.3
SGD	96	98	97	98	99	98	97	96	98	97	97.93
RC	97	98	97	98	97	98	98	97	96	97	97.92
SDGR	98	98	97.5	98	97.3	98.8	98	99	98	97.6	98.02

### Ablation study

We conducted an ablation study at the algorithm level to examine the impact of every component and demonstrate the contribution of the proposed SGDR system. To experiment, we utilized the proposed string processing algorithm, both with and without it, in traditional and proposed models. Initially, we performed classification experiments without the string processing algorithm. Subsequently, we trained the models with the proposed string processing algorithm. The results of the ablation study are depicted in Table 12. Regarding classification accuracy, the experimental results show that SGDR outperforms other models in both cases, with and without the string processing algorithm. SGDR achieved 97.98% accuracy without the string processing algorithm, while using the proposed string processing algorithm, it achieved 98.12% accuracy.

**Table 12:** Ablation study.

Model	Performance without string processing algorithm	Performance with string processing algorithm
SGD	97.29	97.79
RC	97.92	98.08
Blending SGDR	97.98	98.12

## Discussion

To ascertain the sentiment of the text, we employ the TextBlob NLP API, which is capable of determining the polarity of the text and aiding in identifying whether the sentiment is negative, positive, or neutral. We select three sentences from the BBC dataset and analyze the frequency of each word. Following the individual calculation of TF and IDF, we arrive at the ultimate TF-IDF value. Subsequently, we gauge the sentences’ polarity based on the TF-IDF value. The first and third sentences exhibit neutral sentiment, with a polarity of 0.0, while the second sentence conveys negative sentiment, with a polarity of -0.2.

Shifting our focus to news categorization, we commence by training the benchmark ML algorithms using the training dataset. The performance is then grouped and tabulated, visualized using box plots. Among supervised ML algorithms, the group encompassing regularization and optimization algorithms (SGD and RC) demonstrates superior performance compared to other algorithms. This group achieves an accuracy of 97.92% along with commendable precision, recall, and f1 score. In distance-based algorithms, SVM attains an accuracy of 97.08%, whereas KNN reaches 91% accuracy. Probability-based LR and NB achieve accuracies of 96.32% and 95.10%, respectively. However, a distinct scenario emerges in tree-based algorithms, where DT exhibits a mere 85.59% accuracy, and AB displays 78.23% accuracy, accompanied by subpar scores in other performance measures.

Conversely, within the domain of unsupervised ML algorithms, NMF shows a 96% accuracy, while KMC achieves a 94% accuracy, both of which are commendable. These algorithms excel in accurately categorizing unlabeled news. After this implementation, we apply our proposed string-processing techniques to the texts. The performance of all algorithms improves significantly when the processed text is employed individually for each algorithm. The pinnacle of accuracy in news categorization is reached when utilizing our proposed ensemble model, SGDR. This model boasts an impressive accuracy of 98.12%, surpassing all other algorithm performances, thus establishing the superiority of our proposed methodology. We also evaluate the superiority of the proposed model using the McNemar significant test, which is more significant than existing benchmark algorithms. The 10-fold cross-validation result shows the algorithms’ stability and no overfitting of the models during classification. The Python code of this work is available in the GitHub repository [65].

The proposed SGDR ensemble model combines the strengths of SGD and RC, offering a robust, efficient, and scalable solution that addresses the limitations of existing methods. Its ability to generalize, prevent overfitting, and handle diverse datasets makes it superior to traditional models like SVM, LR, KNN, NB, DT, AB, and others. The SGD component in the SGDR model is responsible for its efficiency and ability to handle diverse datasets. It updates the model parameters incrementally for each training sample, which makes it faster and more scalable. On the other hand, the RC component applies L2 regularization in the proposed SGDR, which helps prevent overfitting by penalizing large coefficients. This L2 regularization helps balance bias and variance, ensuring the SGDR generalizes well to unseen data. This is often a challenge with models like DT and KNN, which can easily overfit. SGD’s ability to update weights incrementally ensures that the model adapts and learns continuously. By blending the ensemble process, SGDR can leverage the strengths of multiple models, potentially achieving better performance than single models like NB and DT, which may not effectively capture the complexities of the data. The inclusion of Ridge regularization within the SGDR model makes it robust to noisy data and multicollinear. This is an advantage over models like KNN and DT, which can be significantly affected by noisy data and feature correlations.

Fake topics can significantly impact the performance of ML classifiers by introducing noise and misleading patterns in the data. These fabricated topics may lead classifiers to learn incorrect associations, reducing accuracy and reliability. Moreover, fake topics can distort the underlying relationships between features, affecting the model’s ability to generalize to unseen data. Detecting and filtering out fake topics is crucial to maintaining the integrity of ML algorithms and ensuring accurate classification results in various domains.

## Conclusion

This work assesses sentiment in news articles and categorizes them using ML algorithms. The study aims to identify a classifier that optimizes news classification accuracy within the ML framework. Employing unsupervised ML algorithms for unlabeled data, the research segments the algorithm into multiple parts, documenting and visualizing their performances through various charts. Utilizing the TextBlob Python library, sentiment analysis based on user opinions is conducted to discern positive, negative, and neutral sentiments from textual content, enhancing comprehension of social news. A blended ensemble algorithm named SGDR is introduced for news categorization, exhibiting a superior accuracy of 98.12%. String processing techniques enhance algorithmic classification efficacy. The McNemar test demonstrates SGDR’s superiority over other algorithms, underscoring its efficacy in news categorization. By adopting this system, organizations can gather and analyze user opinions to refine products and services based on public sentiment, enabling them to monitor pivotal messages derived from public opinions and perceptions concerning shared news.

This study has limitations stemming from a small dataset, restricting the generalization of findings. Real-time data constraints may impact the study’s timeliness, as it relies on historical information. Additionally, the choice of a less complex model, while practical for computational efficiency, may limit the model’s ability to capture intricate patterns in more sophisticated systems. These limitations underscore the necessity of cautious interpretation and consideration when applying the study’s results to broader contexts, emphasizing the need for future research with larger datasets, real-time data integration, and exploration of more complex models for comprehensive insights.

## Future work

Future work could focus on enlarging datasets, integrating real-time data streams, and exploring more sophisticated models to address limitations. Subsequent research endeavors will include multiple newspapers for sentiment analysis and expanding news categories. Furthermore, the relationship between news articles over an extended period could be explored in forthcoming investigations.
